# Supplementary material for: Early outcome prediction with quantitative pupillary response parameters after out-of-hospital cardiac arrest: A multicenter prospective observational study​
Source: PLoS One. 2020 Mar 19;15(3):e0228224. doi: 10.1371/journal.pone.0228224 (PMC7082023; doi:10.1371/journal.pone.0228224)
Supplement: S1 Table — CV, constriction velocity; DV, dilation velocity; IQR, interquartile range; LAT, latency of constriction; MAX, maximum diameter; MCV, maximum constriction velocity; MIN, minimum diameter; NPi, neurological pupil index. (DOCX) [file pone.0228224.s001.docx]

S1 Table. Median values of quantitative pupillary parameters

|  | Favorable  neurological outcome (N = 13)  Median [IQR] | Unfavorable  neurological outcome (N = 37)  Median [IQR] |
| --- | --- | --- |
| MAX (mm) 0h  6h  12h  24h  48h  72h | 3.4 [2.9–4.2]  3.2 [2.5–4.4]  2.7 [2.2–4.1]  3.1 [3.1–3.8]  2.5 [2.1–3.8]  2.6 [2.2–3.6] | 4.6 [1.9–5.9]  3.3 [1.9–5.3]  3.5 [2.1–5.2]  3.7 [2.2–5.0]  4.2 [2.4–4.9]  4.8 [2.7–5.4] |
| MIN (mm) 0h  6h  12h  24h  48h  72h | 3.9 [2.2–5.6]  2.2 [2.0–3.8]  2.0 [1.8–3.4]  2.1 [1.8–2.6]  2.1 [1.8–2.1]  2.2 [1.8–2.6] | 4.8 [2.7–5.4]  5.0 [3.4–6.4]  4.2 [2.1–5.5]  3.6 [2.2–5.5]  2.5 [1.9–4.2]  3.0 [2.1–4.7] |
| CV (mm/sec) 0h  6h  12h  24h  48h  72h | 0.9 [0.6–2.8]  1.3 [0.6–2.5]  1.2 [0.4–2.2]  1.2 [0.6–1.8]  1.1 [0.2–2.4]  1.1 [0.9–1.6] | 0 [0–0.2]  0.2 [0–1.0]  0.3 [0–1.1]  0.1 [0–1.4]  0.3 [0–1.1]  0.3 [0–1.2] |
| MCV (mm/sec) 0h  6h  12h  24h  48h  72h | 1.4 [0.8–2.9]  2.2 [0.8–3.6]  1.4 [0.8–3.8]  1.6 [0.6–2.7]  1.6 [1.2–3.5]  1.8 [1.1–2.3] | 0 [0–0.7]  0.4 [0–1.1]  0.4 [0–1.3]  0.2 [0–2.1]  0.7 [0–1.8]  0.5 [0–2.0] |
| DV (mm/sec) 0h  6h  12h  24h  48h  72h | 0.4 [0.2–1.0]  0.8 [0.2–1.1]  0.4 [0.2–1.0]  0.6 [0.1–0.7]  0.9 [0.3–1.2]  0.5 [0.4–0.8] | 0 [0–0.2]  0.1 [0–0.3]  0.2 [0–0.8]  0.1 [0–0.6]  0.1 [0–0.6]  0.1 [0–0.6] |
| LAT (msec) 0h  6h  12h  24h  48h  72h | 0.3 [0.3–0.5]  0.3 [0.3–0.3]  0.3 [0.3–0.3]  0.4 [0.3–0.4]  0.3 [0.3–0.4]  0.3 [0.3–0.3] | 0.4 [0.3–0.5]  0.3 [0.3–0.4]  0.4 [0.3–0.5]  0.3 [0.3–0.4]  0.3 [0.3–0.4]  0.3 [0.3–0.4] |
| NPi 0h  6h  12h  24h  48h  72h | 4.1 [3.5–4.4]  4.5 [3.8–4.8]  4.4 [4.2–4.8]  4.3 [3.9–4.6]  4.6 [4.3–4.6]  4.5 [4.1–4.8] | 0 [0–3.5]  3.3 [0–4.3]  3.7 [0–4.6]  3.4 [0–4.5]  3.7 [0–4.6]  3.5 [0–4.5] |

CV, constriction velocity; DV, dilation velocity; IQR, interquartile range; LAT, latency of constriction; MAX, maximum diameter; MCV, maximum constriction velocity; MIN, minimum diameter; NPi, neurological pupil index.
