# Supplementary material for: Early outcome prediction with quantitative pupillary response parameters after out-of-hospital cardiac arrest: A multicenter prospective observational study​
Source: PLoS One. 2020 Mar 19;15(3):e0228224. doi: 10.1371/journal.pone.0228224 (PMC7082023; doi:10.1371/journal.pone.0228224)
Supplement: S1 Fig — Values represent area under the curve [95% confidence interval]. LAT, latency of constriction; MAX, maximum diameter; MIN, minimum diameter. (DOCX) [file pone.0228224.s003.docx]

**A**

**B**

**C**

S1 Fig. Serially measured quantitative pupillary responses of favorable and unfavorable neurological outcomes after return of spontaneous circulation to 72 h.

Values represent area under the curve [95% confidence interval]. MAX, maximum diameter; MIN, minimum diameter; LAT, latency of constriction.
