# Supplementary material for: Early outcome prediction with quantitative pupillary response parameters after out-of-hospital cardiac arrest: A multicenter prospective observational study​
Source: PLoS One. 2020 Mar 19;15(3):e0228224. doi: 10.1371/journal.pone.0228224 (PMC7082023; doi:10.1371/journal.pone.0228224)
Supplement: Attachment — Submitted filename: Response_to_Reviewers_1128(JN).docx [file pone.0228224.s004.docx]

**Point-by-point response to reviewer comments**

Thank you for the constructive suggestions for improving our original manuscript. Our manuscript has benefited from these insightful suggestions. Our point-by-point response to each comment is written as plain text below the reviewer’s points *(italics).*

**Journal Requirements:**

***1. When submitting your revision, we need you to address these additional requirements.***

***Please ensure that your manuscript meets PLOS ONE's style requirements, including those for file naming. The PLOS ONE style templates can be found at***

***http://www.journals.plos.org/plosone/s/file?id=wjVg/PLOSOne_formatting_sample_main_body.pdf and http://www.journals.plos.org/plosone/s/file?id=ba62/PLOSOne_ formatting_sample_title_authors_affiliations.pdf***

Reply: We have confirmed that our manuscript meets PLOS ONE’s style requirements.

***2. Thank you for including your ethics statement:***

***"This study is in adherence with ethical guidelines and approved by IRB of each participating institution. Written informed consent was obtained from patients’ family as appropriate. ".***

***a. Please amend your current ethics statement to include the full name of the ethics committee/institutional review board(s) that approved your specific study.***

Reply: The names of the ethics committee/institutional review boards have been added in Methods, Study design and setting section, as described below (Page 4, Lines 79-82):

This study was approved by the IRB of each participating institution (Keio University School of Medicine, Ethics Committee; Institutional Review Board of Nippon Medical School; Research Ethics Committee, Tokyo Saiseikai Central Hospital; and Independent Ethics Committee of Hiratsuka City Hospital).

***b. Once you have amended this/these statement(s) in the Methods section of the manuscript, please add the same text to the “Ethics Statement” field of the submission form (via “Edit Submission”).***

Reply: We amended the aforementioned ethics statement in the submission form accordingly.

***3. We note that you have indicated that data from this study are available upon request. PLOS only allows data to be available upon request if there are legal or ethical restrictions on sharing data publicly. For information on unacceptable data access restrictions, please see http://journals.plos.org/plosone/s/data-availability#loc-unacceptable-data-access-restrictions.***

******* ***In your revised cover letter, please address the following prompts:***

***a) If there are ethical or legal restrictions on sharing a de-identified data set, please explain them in detail (e.g., data contain potentially identifying or sensitive patient information) and who has imposed them (e.g., an ethics committee). Please also provide contact information for a data access committee, ethics committee, or other institutional body to which data requests may be sent.***

***b) If there are no restrictions, please upload the minimal anonymized data set necessary to replicate your study findings as either Supporting Information files or to a stable, public repository and provide us with the relevant URLs, DOIs, or accession numbers. Please see http://www.bmj.com/content/340/bmj.c181.long for guidelines on how to de-identify and prepare clinical data for publication. For a list of acceptable repositories, please see http://journals.plos.org/plosone/s/data-availability#loc-recommended-repositories.***

***We will update your Data Availability statement on your behalf to reflect the information you provide.***

Reply: We have uploaded a de-identified data set to the repository (UMIN-ICDR). The data set can be found at the following URL:

https://upload.umin.ac.jp/cgi-bin/icdr_e/ctr_view.cgi?recptno=R000018164

Reviewer #1:

***1. Could the study authors comment on why the 0 hour measurements were the only measurements found to significantly impact the ROCs?***

Reply: Thank you for pointing an interesting point. We believe that this is accounted for by two reasons. Although the change over time in both outcome groups were not statistically different, in parameters that yielded high prognostic power (see Figure 1 C, D, E, G), the poor outcome group showed the lowest values at 0 h; thus, the differentiation in each parameter between the favorable group was best at 0 h. Another reason is related to the nature of cardiac arrest research, i.e., the number of analyzed patients decrease as patients die over time. Thus, a decrease in sample size decayed the prognostic power. Further large-scale study will confirm our findings. In addition, the small sample size is stated as a limitation (Page 14, Line 311).

***2. I find the heterogeneity of the patient characteristics and small sample size problematic in being able to exclude patient characteristics as a main driver in the quantitative pupillary response differences at time 0. The authors comment on*** ***pupillary response differences based on age, drugs used during resuscitation and therapeutic hypothermia. Could the authors comment on why the differences noted in pupillary responses between the two groups are not accounted for by the differences in patient characteristics?***

Reply: Thank you for an interesting question. Patient characteristics, patient demographics, as well as information regarding resuscitation are different among favorable and unfavorable neurological outcome groups. These are well-established associations in cardiac arrest research. As you pointed out, one may argue that baseline differences, particularly in pupillary response parameters prior to cardiac arrest, are augmented by cardiac arrest and resuscitation. Since it is impossible to obtain pupillary response parameters before cardiac arrest, the influence of different patient demographics on pupillary light responses cannot be determined in cardiac arrest research.

Latency is reported to prolong with age. Although our poor neurological outcome group was composed of older patients, latency was not different between the two outcome groups in our cohort. This could imply that pupillary responses after cardiac arrest might not only reflect the patient demographics before cardiac arrest.

Drugs used in post-resuscitation care are provided in the table below. None of the drug usage was statistically different among both groups; thus, we think that 0-hour pupillary responses were not accounted for by drugs. Similarly, none of the patients were hypothermic at 0 h; thus, TTM had no influence on 0-hour pupillary response parameters.

Reviewer only Table 1. Usage of specific pharmacologic agents among 90-day neurological outcome groups at 0 h

| Pharmacologic agents | Favorable neurological outcome (N = 13) | Unfavorable neurological outcome (N = 37) | P value* |
| --- | --- | --- | --- |
| Propofol; n (%) | 0 | 8 (22) | 0.09 |
| Midazolam; n (%) | 0 | 0 | **-** |
| Dexmedetomidine; n (%) | 0 | 0 | - |
| Fentanyl; n (%) | 0 | 4 (11) | 0.56 |
| Buprenorphine; n (%) | 0 | 0 | - |
| Norepinephrine; n (%) | 1 (8) | 9 (24) | 0.26 |
| Dopamine; n (%) | 2 (15) | 16 (43) | 0.07 |
| Dobutamine; n (%) | 1 (8) | 2 (5) | 0.60 |

* Fisher’s exact test.

***3. Could the authors also clarify the statement on page 8 line 168-171: "Similar to PLR (P=0.86)[9], after accounting for the interaction at a certain time point by outcome status, none of the pupillary parameters were significant...". If this statement refers to the fact that there was no difference in change of pupillary parameters over time regardless of outcome, then the statement would need to be reworded to reflect this.***

Reply: Thank you for this comment. We agree that this statement is unclear. We removed redundant statements and reworded this sentence as follows (Page 9, Lines 168-176):

CV (P < 0.001), MCV (P < 0.001), DV (P < 0.001), and NPi (P = 0.005) as well as PLR (P < 0.001) [9] were consistently greater among patients with favorable neurological outcomes than those with unfavorable outcomes at each time point during the first 72 h. On the contrary, no difference in MAX, MIN, and LAT was observed between the two outcome groups. Similar to PLR (P = 0.86) [9], after accounting for the interaction at a certain time point by outcome status, none of the pupillary parameters were significant (MAX, P = 0.17; MIN, P = 0.86; LAT, P = 0.82; CV, P = 0.39; MCV, P = 0.63; DV, P = 0.79; and NPi, P = 0.91). This indicates that there is no difference in the change of each pupillary parameter over time regardless of the outcomes (Fig 1, S1 Table).

***Reviewer #2:***

***1. General comments: While outcome prediction from cardiac arrest using pupillometry is no longer a novel concept, this study adds important data regarding 1) optimal timing of the tool and 2) thorough assessment of alternate predictors. These data will be useful as the tool becomes more prevalent at the bedside. In this way, the study will be of interest to PLOS ONE readers. However, a key limitation of the study that the authors should address is the lack of transparent and detailed reporting of patient CPC, which will affect how their results should be interpreted.***

Reply: Thank you for the comment. We agree that the breakdown of each CPC category is important; we therefore added them in Table 1 (Pages 7-8). There was only one patient with CPC 3 in our cohort. Our point-by-point response to your comments is shown below.

***2. Methods: Why did you choose to include non-comatose*** ***post-CA patients? To reference your introduction, the literature for PLR is in comatose post-CA patients. The description of the protocol (line 94 to end of paragraph) also*** ***leads the reader to think this study was conducted in comatose patients; please clarify the language.***

Reply: There is no argument that neuroprognostication for comatose post-cardiac arrest patients is the center of clinical interest, because non-comatose post-cardiac arrest survivors generally achieve favorable neurological outcomes. We designed this study to test the prognostic performance of quantitative parameters in post-cardiac arrest patients and included all eligible patients irrespective of their consciousness level. Although all non-comatose patients in our cohort achieved favorable neurological outcome, we wondered if it would produce bias by excluding non-comatose patients who failed to achieve good neurological outcome. As suggested, we revised the description of the protocol (Page 5, Line 96-105) so that the readers would not think that this study was conducted in comatose patients:

Comatose (GCS ≤8 with motor response of ≤5) patients received standard post-arrest care according to international guidelines [4, 15]. … Non-comatose (GCS >8 in the ED) post-CA patients were also enrolled but did not undergo therapeutic hypothermia [9].

***3. Analysis: For the selection of potential confounders, did you include CPR duration? It is not listed in line 136. Was the “a priori knowledge” of selected clinical predictors based on*** ***clinical expertise or a prior logistic regression model? Please expand.***

Reply: Thank you for raising an important point. We did not include the duration of CPR as a covariate in our multivariable logistic regression model. Because 26 (52%) patients had an unwitnessed arrest and 14 (28%) patients had a witnessed arrest but did not receive bystander CPR, the duration of CPR did not indicate the severity of anoxic brain damage in our series. We selected the potential confounders based on previously published literatures (refs #4, 18-20). We did not utilize a commonly used method of combining univariable analysis and further incorporating variables that shows P value threshold less than 0.1 into the multivariable model. To improve clarity, we revised the description about the selection of the potential confounders, as shown below (Page 6, lines 139-141):

A set of potential confounders was chosen as clinical predictors based on published literature including witness status, bystander CPR, initial shockable rhythm, and implementation of TTM [4, 18–20].

***4. Results: Please report the breakdown of CPC. How many patients were in each CPC category, (i.e., 1, 2, 3, 4, 5)? This is relevant because your study’s two outcome groups could be weighted towards one extreme. For example, the “unfavorable neurologic outcome” group could be mostly CPC 5 (brain death) or perhaps CPC 3 (severe but conscious). These are clearly two very different phenotypes. I would interpret the study differently depending on the breakdown of the groups. Also, it is standard to report CPC subgroups. Additionally, for quantitative measurements of pupillary responses (line 163), please report median values and IQR in the text, or include these values as a supplemental table.***

Reply: Thank you. We added the number of patients in each CPC category in Table 1 (Page 8). We added a supplemental table reporting the median values along with the IQR of the quantitative pupillary response parameters (Supplementary materials, P2-3).

***5. Discussion: Were there any outliers? It may be useful to discuss if there were patients who had a low pupillary response but who still had a favorable neurologic outcome.***

Reply: Thank you for this question. Median values and interquartile ranges are provided in Supplemental Table 1. Using 1.5 times the interquartile range beyond Q1 and Q3 to define an outlier, no measurements corresponded to an outlier.

***Regarding line 319, I think you mean to say “Japanese physicians DO NOT withhold aggressive treatments.” It is important to mention that the study was unblinded, and I think while the cultural insights here are very useful to the reader, the language in the last sentence line 319 reads a bit too confident.***

Reply: Even if poor neurological prognosis is perceived, physicians in Japan seldom selected active WLST from cultural preferences. Thus, mortality did not concentrate within a week after CA and continued to increase over 3 weeks, and 16% of our patients achieved CPC of 4 at 90 days, which is distinctly different from those in other countries. We agree that an unblinded protocol is a major limitation of our study; we therefore rephrased the following passage (Page 15, Lines 321-324):

Japanese physicians seldom adopt active WLST even if poor neurological prognosis is perceived. As mortality continued to increase over 3 weeks and did not concentrate in the first week [9], we consider that unblinded results have had small effect on self-fulfilling prophecy.

***6. Conclusions: This is alphabet soup and a bit too granular. You might start the paragraph by saying “several additional parameters were associated with favorable neurologic outcome, including XYZ.” The final sentence line 325 is confusing and not grammatically correct and needs to be reworked.***

Reply: As suggested, we have revised the Conclusions as follows (Page 15, Lines 327-333):

Several additional parameters were associated with favorable neurologic outcomes including CV, MCV, DV, and NPi, similar to PLR. These parameters were consistently greater…We hope that future studies will reveal the utility of quantitative values at 0-hour pupillary response, especially PLR as a parameter for multimodal prognostication.

***7. Figures: Figure 1: please show subgroups (n=x) of CPC. It appears there is overlap between groups (e.g., frames C, D, E, F, G) but the SEM are not reported in the text. Please specify in the results section. General comments: would be helpful for the reader if you label each panel more clearly.***

Reply: The number of each subgroup was added to the caption of Figure 1. In the legends of Figure 1, we added the description that data were expressed as mean ± SD (P9, Lines 183-184) and modified the labels of Figures 1, 2, and 3 to enhance visibility.

***8. Tables: Please report CPC subgroups.***

Reply: The 90-day CPC breakdown is added to Table 1.
